# Quality of Life of Adolescents and Young Adults After Testicular Prosthesis Surgery During Childhood: A Qualitative Study and Literature Review

**DOI:** 10.3390/children12060720

**Published:** 2025-05-31

**Authors:** Zoi Chantzi, Sotirios Fouzas, Alexandros Drivalos, Athanasia Stamati, Agapios Gkentzis, Maria Athanasopoulou, Katerina Kambouri, Despoina Gkentzi, Eirini Kostopoulou, Anastasia Vareli, Evangelos Blevrakis, Konstantinos Zachos, Vasileios Alexopoulos, Antonios Panagidis, Panagiotis Plotas, Aspasia Louta, Ageliki A. Karatza, Theodore Dassios, Gabriel Dimitriou, Eleni Jelastopulu, Xenophon Sinopidis

**Affiliations:** 1 Department of Pediatric Surgery, University Hospital, 26504 Patras, Greece; up1098156@upatras.gr (Z.C.); a_louta@upatras.gr (A.L.); 2 School of Medicine, University of Patras, 26504 Patras, Greece; sfouzas@upatras.gr (S.F.); gkentzid@upatras.gr (D.G.); irekost@upatras.gr (E.K.); karatza@upatras.gr (A.A.K.); tdassios@upatras.gr (T.D.); gdim@upatras.gr (G.D.); 3 Department of Pediatrics, University Hospital, 26504 Patras, Greece; 4 Department of Urology, General Hospital of Ilia, 27100 Pyrgos, Greece; alexdriv@yahoo.gr; 5 Department of Psychiatry, General Hospital of Ilia, 27100 Pyrgos, Greece; sasha_stamati@yahoo.gr; 6 Department of Urology, Royal Bolton Hospital, Farnworth, Bolton BL40JR, UK; agapiosgkentzis@hotmail.com; 7 Department of Pediatric Surgery, Children’s Hospital, 26331 Patras, Greece; mathanaso@upatras.gr (M.A.); a.vareli@karamandaneio.gov.gr (A.V.); zachos.i.k@gmail.com (K.Z.); v.alexopoulos@karamandaneio.gov.gr (V.A.); a.panagidis@karamandaneio.gov.gr (A.P.); 8 Department of Pediatric Surgery, Democritus University of Thrace, 69100 Alexandroupolis, Greece; kampouri@med.duth.gr; 9 Department of Pediatric Surgery, University Hospital, 71003 Heraklion, Greece; e.blevrakis@uoc.gr; 10 School of Medicine, University of Crete, 71003 Heraklion, Greece; 11 Department of Speech and Language Therapy, School of Health Rehabilitation Sciences, University of Patras, 26504 Patras, Greece; pplotas@upatras.gr; 12 Department of Public Health, School of Medicine, University of Patras, 26504 Patras, Greece; jelasto@upatras.gr

**Keywords:** testicular prosthesis surgery, pediatric, children, quality of life, physical health, mental health, self-esteem, self-confidence, interpersonal relationships, sexuality

## Abstract

Background/Objectives: To evaluate the quality of life (QoL) of adolescent and young adult males who had testicular prosthesis surgery during their childhood, as well as their own and their parents’ inner perceptions and feelings. To our knowledge, this is the first qualitative study and one of few with an integrated approach on the QoL of pediatric testicular prosthesis recipients. Methods: Recipients and their parents were interviewed regarding their life with the testicular prosthesis. Major QoL domains (i.e., physical and mental health, interpersonal relationships, sexuality) were assessed. Results: Although there were participants who viewed the outcomes with negativity, most expressed satisfaction. Daily routine returned to normal after a postoperative period with precautions, such as fear of damaging either the implant or the healthy testis. Prosthesis feeling was acceptable and normalized with time, while self-image and self-confidence were boosted by the presence of the prosthesis. All participants outlined the importance of privacy as being kept in the inner family circle. Some exhibited introverted behavior. Regarding sentimental and sexual intimacy, the prosthesis produced anxiety and concern both in experienced recipients and minors, which was moderated by a normal scrotal appearance. Conclusions: Testicular prosthesis surgery performed from childhood to adulthood may create profound and variable concerns, which may affect the recipients and their companions in the long term. Therefore, it should not be considered in a simplistic manner as a routine procedure and should be preceded by thorough conversation with experts.

## 1. Introduction

Testicular absence in children, often resulting from congenital conditions (e.g., anorchia, cryptorchidism), testicular torsion, trauma, or orchiectomy for cancer, can profoundly affect body image and psychosocial well-being during the critical developmental stages [1]. Among the causes, testicular torsion is the leading factor in over 80% of pediatric cases of testicular loss, and timely recognition and intervention, preferably within six hours, can preserve testicular viability in up to 90–100% of cases. If treated within 6–12 h, depending on the degree of the torsion, 20–50% of testes will be saved, and if treated within 12–24 h, only 0–10% of testes will be saved [2]. Studies on the long-term effects of orchiectomy showed an emergence of psychological issues both in boys and adults, as the absence of one or both testes is considered as a major risk factor for affecting their psychosexual development [3,4,5].

The quality of life (QoL) of young males after testicular prosthesis surgery during childhood has not been thoroughly and holistically studied, in relation to anatomical, biological, and functional postoperative parameters [6,7]. Those who have mainly attracted scientific interest so far were mainly adults after orchiectomy due to testicular cancer. Pediatric-specific QoL data remain limited and most related published articles have focused on their respective physical outcomes, especially complications rather than integrated QoL measures [8,9,10].

Although traditional medical research is quantitative in nature, here, a qualitative methodology was used [11,12,13,14,15]. This approach has been underutilized, and somewhat snubbed in the past by biomedical sciences, medicine being amongst them. However, starting with studies in psychology, qualitative research has proven to increase the power of the results of clinical trials [16]. Such studies have already been undertaken in pediatric urology [17,18]. Although there is currently a lack of clear guidelines for reporting qualitative research, there has been a recent effort to organize such strategies [19].

The aim of this study is to use a qualitative approach on the QoL of adolescent or young adult males who underwent testicular prosthesis surgery during childhood, recording their overall experience regarding their general health in relation to physical, psychological, social, and sexual outcomes, and the perceptions and well-being of their parents. As Papamichail and Bates have quoted, it is anticipated to “allow their voices to be heard” [20].

## 2. Materials and Methods

### 2.1. Study Population

This qualitative study included males who as children underwent testicular prosthesis implantation surgery from 1 January 2010 to 31 December 2023, and their parents. The participants were inhabitants of the region of Western Greece and Peloponnese, an area corresponding to a population of about one million inhabitants. The inclusion criteria for the participation in the study were as follows: age under eighteen years at the time of operation, absence of neurodevelopmental issues, no recent divorce or death in the family, available pharmaceutical history, no cancer history, acquisition of written informed consent for adult participants, and parental consent and child assent for minors. Fluent knowledge of the Greek language was considered essential for optimal understanding and avoiding misleading statements during the interview. As QoL with a testicular prosthesis was the main topic of this project, we investigated the main sections that might have possibly been affected in such patients, namely physical and mental health, interpersonal relationships, family communication, access to information, and sexuality.

### 2.2. The Operation and the Prosthesis Material

**Figure 1:**
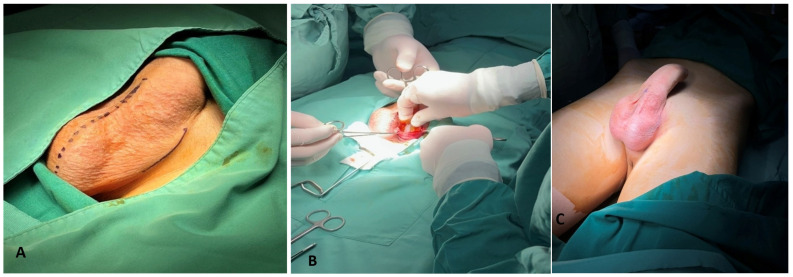
(**A**) The initial drawing of the incision in the crease between the root of the penis and the groin in a 12-year-old patient with an empty left hemiscrotum (continuous line). The dotted line is the scrotal raphe on the midline. (**B**) The testicular prosthesis is implanted in the created subdartos pouch. (**C**) The esthetically acceptable result at the end of the operation, with the testicular prosthesis implanted in the left hemiscrotum.

The operations for the implantation of the testicular prostheses were performed by different pediatric surgeons at the Children’s Hospital of Patras. The implant was of silicon elastomer, aimed to provide a natural consistency and feeling to the patient. The solid silicone prevented failure of leakage or loss of shell integrity (Promedon GMBH International, Kolbermoor, Germany). The surgical approach to the empty hemiscrotum was effected through the crease between the root of the penis and the groin (Figure 1A). The silicone prosthesis was implanted in a created subdartos pouch (Figure 1B). This resulted in an esthetic outcome without any evident scar as the surgical wound was hidden in the crease (Figure 1C). The duration of surgery was about 25 min, and the patient returned home the same day.

### 2.3. The Interview

The method which was considered appropriate for this study was that of a semistructured interview. Though the content was mainly set by the participants, it was supported by a questionnaire consisting of opened-ended questions developed by the research team in Greek and translated in English by the authors of this research. The questionnaire was not validated (Table 1).

The questions were not formatted in the shape of a conventional questionnaire but served as a supporting guide, a compass for the interview. This structure was felt to be more useful, especially for the younger participants. Jaakola has stated that a semi-constructed interview in an onsite setting is preferred even for children under the age of ten years [21]. The questions were used at the researcher’s discretion to reach a balanced acquisition of information provided by the participants; the aim was to encourage a spontaneous flow of communication during the interview, without strict adherence to the questions, making the participant feel relaxed and free to express his thoughts and feelings. Herein, as stated in a definition of qualitative methodology, credibility was used instead of validity, dependability instead of reliability, and conformity instead of objectivity [22].

All interviews were conducted by the same researcher from 1 September 2022 to 31 January 2024. In the first contact with the patients and/or parents over the phone, the researcher explained the aim of the study and arranged face-to-face appointments at locations of the patients’ or parents’ own choice, such as at home, a coffee shop, their workplace, a hospital office, or via internet teleconference, aiming for the most comfortable setting for the participants.

The participants were informed about the study’s details and signed the appropriate informed consent documents. They were also informed that they could quit the process anytime they wished, either during or after the interview. Anonymity was reassured as well. All conversations were recorded, to guarantee the accuracy of information during analysis. Every participant was interviewed separately, without the presence of others. They were also reassured that there were no right or wrong answers, and that the researcher was interested in obtaining their own experience, with particular interest in those features they considered most important. Given the sensitive and emotionally charged nature of the interview content, including handling assent and consent, and addressing minors, some of them in the sensitive age between eleven and fourteen years, the interviewing researcher attempted to provide psychological assistance if needed.

### 2.4. Data Analysis

Debriefing and interpretation of the recorded files followed. Thematic analysis resulted in the classification of the acquired information, and the development of domains, thus reaching a systematic presentation of the acquired information [23]. The domains studied included self-evaluation of the subject’s physical status (i.e., pain or discomfort, body functional level, feeling of the prosthesis), psychological wellness (self-esteem, anxiety, adaptation), social evolution (overall satisfaction of the participant’s life course, participation in social activities, social role performances, interpersonal relationships, support by his inner-circle family members and friends), and finally sexuality (satisfaction of sex life as a recipient of a testicular prosthesis, and for the younger ones without sexual experiences, perception on the effect of the prosthesis on their future intimate life). All outcomes were encoded and adjusted to the mentioned domains. The outcomes were associated with the observations of the researcher who performed the interviews.

### 2.5. Outcomes of the Study

In this study we anticipated uncovering the recipients’ inner feelings, worries, perceptions, regrets, advances, and setbacks during the years following surgery, in each section of their lives. At the same time, we expected to discover their parents’ own perspectives, particularities, and diversities compared to those of their sons. The major anticipated outcome was discovering the optimal way of dealing with the effects on QoL after testicular prosthesis implantation surgery at the sensitive age of childhood and adolescence.

## 3. Results

### 3.1. Study Population Characteristics

A total of 44 males received a testicular prosthesis as children from January 2010 to December 2023. Contact was not possible for 15 of them, either because of missing contact information or due to participants’ lack of response to the researcher. Thirteen more recipients declined to participate or withdrew consent. Thus, the final study population included 16 subjects with an age range of 11–27 years (mean 19.12 years) at the time of the interview, while their age at the time of surgery ranged from 8 to 16 years (mean 12.75 years). The interval between operation and contact ranged from 1 to 13 years (mean 6.37 years) (Table 2).

The causes which resulted in orchiectomy and consequently in testicular prosthesis surgery included atrophic undescended testes (*n* = 6), testicular torsion (*n* = 5), in situ testicular atrophy (*n* = 3), anorchia (*n* = 1), and hypogonadism (*n* = 1). Fourteen recipients had unilateral prostheses, and two had bilateral prostheses. Nine recipients were adults, and seven were children or teenagers (under 18 years) at the time of the interview. Mothers (*n* = 6, age 44–50 years) and fathers (*n* = 6, age 46–54 years) of the recipients participated in the survey as well (Table 3).

The interviews were performed at home (*n* = 5), in a healthcare service office (*n* = 4), in an external location (*n* = 3), work (*n* = 2), or through internet teleconference (*n* = 2). Each interview lasted 20–40 min for the adults and 15–20 min for the minors, respectively. An introductory 15 min was effective to obtain consent and create an environment of acceptance and confidence. The overall time the participants and their family spent with the researcher lasted between 70 and 120 min, with a mean of 90 min.

### 3.2. Quality of Life Domain Outcomes

The outcomes of the interviews were debriefed and classified into six domains, subdivided into 14 codified sections of interest (Table S1 in Supplementary Materials).

#### 3.2.1. Physical Health

Feeling of the testicular prosthesis

Almost all recipients (*n* = 15) reported that the feeling of the testicular prosthesis was different from that of the normal testis, describing it as tougher, heavier, and located higher. R_12_ reported that it produced a colder effect, while R_01_ avoided touching it. Nevertheless, most recipients reported that they got used to the new sensation with time.

Mothers reported that their sons felt uncomfortable with the prosthesis, because of the difference from the normal testis and a tougher impression. M_R15_ completely avoided any relevant discussion with her son, while M_R04_ was unaware that her son hid negative feelings. In general, they avoided these discussions with their sons. Three fathers presumed that their sons were satisfied with the prosthesis, though they never discussed it with them. F_R04_ used to palpate the prosthesis while his son was asleep, unsatisfied by the feeling, while F_R15_ avoided discussing it with his son who had already openly discussed his experience.

Restriction of activities

All recipients reported restriction of their daily activities during a postoperative period lasting from a few days to five months. Beyond this period, they considered that it did not affect them significantly. They presented a mildly higher vigilance to avoid trauma. Actually, the fear of injury of the healthy testis was greater than that of damaging the prosthesis. R_05_ used pillows when seated, two years after surgery. All mothers arbitrarily extended the period of constricted life activities against medical instructions. When normality returned, there was still vigilance, especially during sports (M_R05_ and M_R12_ revealed that their sons used protective testicular devices). M_R12_ reported that the testicular prosthesis was disturbed after prolonged swimming. Only M_R15_ considered her son free of restrictions after the first postoperative days. Opposite to mothers, all fathers reported complete absence of restrictions, except one who reported moderate guarding during sports.

Perceptions on fertility

Most recipients (*n* = 9) and all parents were aware that fertility was not affected, as long as there was an intact testis. Four were unaware whether the absence of a testis might negatively affect fertility, and this problem did not concern them until the interview occurred. As for the two recipients with bilateral prostheses, they had already come to terms with this condition and were already under testosterone replacement at the time of surgery, having been followed up by pediatric endocrinologists. R_13_ believed that the prosthesis would assist the remaining testis in fertility. F_R02_ said he was not interested in his son’s fertility potential, but only about his health.

#### 3.2.2. Mental Health

Self-image

Most recipients considered that their physical image had been improved. They felt that a problem had been resolved, not being awkward anymore as prior to surgery, especially in circumstances when they were more exposed, such as when wearing swimsuits. Most parents reported they considered their sons confident about their image. A particular family situation regarded R_05_. His mother perceived he was anxious about his body and the effects on his relationship with girls: “which girl will accept a boy who has one natural testicle? what if she touches the implant”, while his father had feelings of guilt because he thought that his son had a delayed hospital admission after he suffered testicular torsion.

Self-respect

Most recipients (*n* = 14) experienced improvement of their self-esteem, reporting a better apprehension of themselves, and having achieved a boost of optimism after surgery. While most mothers (*n* = 5) considered their sons’ self-respect as high, M_R05_ was worried, as her son developed an introverted character. A positive outlook was derived from the paternal interviews as well.

Feelings

Most recipients were not ashamed of their testicular prosthesis, felt well, less exposed, and without major reservations. Certain patients were upset because of comments related to prosthesis. They were also afraid as to whether this would be discovered by others. R_13_ reported anger because his parents discussed it outside the family and was upset when his friends teased him: “when I was wearing shorts during sports, my friends teased me saying, show us how the fake one is”.

The word “relief” was mainly used by the majority of parents as a serious problem was solved. However, there were still feelings of agony about the recipients’ self-esteem, preoccupation about the healthy testis, or concerns about the possibility of a complication or a second operation, while F_R05_ continued to live in agony and doubts, characteristically reporting: “I understand that he suffers deeply, something makes him too worried”.

#### 3.2.3. Interpersonal Relationships

Same-age peers

Of the eleven recipients who commented on this topic, none discussed the presence of a testicular prosthesis with friends of the same age or others. Either they did not consider it as a matter of public discussion, or were afraid that their friends would make negative thoughts about them. Three recipients discussed it with their best friends, while only R_10_ discussed it with more. R_13_ mentioned information leak by their parents to their friends against his will.

Mothers perceived that their sons did not ever discuss the presence of the testicular prosthesis with peers. The parents of R_02_ advised him to keep it secret. Similarly, most fathers were negatively predisposed to any discussion, even with a close friend. Half of the parents discussed the testicular prosthesis surgery with their own closest friends, a fact that comforted them enough. The second group were negatively predisposed to any sharing option, considering it as a matter of strict privacy.

Intimate partner

Eleven recipients reported that they did or would like to discuss the presence of testicular prosthesis with their present or future partner. They all defined as a partner to discuss with a person who would be in a serious relationship with a developed sense of confidence and intimacy, not an occasional erotic adventure. R_06_ and R_08_ reported that they would never discuss this topic even with their permanent intimate partner. Furthermore, R_06_, who was already in an ongoing relationship, without having ever reported the presence of the testicular prosthesis, believed that his companion did not understand it: “my girlfriend did not mention anything about it…I guess, she did not notice it…I think I would not like to share it even with my permanent partner ”.

The mothers of the minor participants considered their sons of an age not appropriate to discuss future relationships. In general, they believed their sons should be open with their partners on this topic at the appropriate time. M_R01_, whose son was in a relationship, believed that he did not discuss this so far, and she had agony about the way that the testicular prosthesis would affect their future together: “how can I find how is he with his girlfriend? I am not curious, I just want to know that everything is fine, how does the girl face it, how does he feel…I believe he did not tell her anything”. By contrast, most fathers supported the option of discussing with the partner, future or present, though they mentioned that it would be ideal if their sons postponed this discussion to a more mature age.

#### 3.2.4. Family Communication

Communicating with family members

Four recipients reported that they were upset, reluctant, and afraid to discuss the presence of a testicular prosthesis with other family members, as they considered that information leakage would negatively affect their lives. R_14_ reported that nothing was discussed in general in his family. By contrast, five recipients preferred to have open discussions in the secure environment of their families. R_13_, who, as was mentioned before, was upset as his parents leaked information to others, attributed this to his mother’s emotional personality: “at times, there was such a mess at home, that I felt sick even if that wasn’t true…sometimes I shouted at her”.

Most parents considered these discussions sufficient as they did with other health matters. If not, it was attributed to other family events such as a divorce (R_01_). Apart from those who considered testicular prosthesis as a matter of the inner family circle, there were cases of information spreading in the extended circle, depending on the particular circumstances of each family.

Communicating consent for surgery with the family

In this subcategory, it was revealed that as children, although almost all recipients (*n* = 14) were informed to a certain degree of the benefits of the testicular prosthesis, which were mainly esthetic and would offer an image of balance in their body, nine recipients were not asked if they desired surgery, as it was considered by their parents as an inevitable option. Only two recipients reported a thorough understanding of the importance of this procedure and consented deliberately after discussing with their parents (R_04_, R_07_). All the recipients would recommend the placement of a testicular prosthesis, except one who expressed concern about future sexual life. As for the parents, they supported the benefits of the operation to their sons, but most of them did not ask if they had any objections prior to surgery.

#### 3.2.5. Access to Information

Most recipients (*n* = 14) reported that they were informed by their doctor in a highly detailed manner and were asked if they desired to proceed to testicular prosthesis surgery. Apart from their doctor and their parents, seven recipients obtained information through the internet, aiming to confirm the validity of the information they had received. R_06_ avoided discussing anything with his parents, as he considered the information provided by his doctor to be enough. It is of note that R_01_ reported that it would be fun if he could hold in his hand the prosthesis prior to surgery, to investigate it. Opposite to the recipients’ perceptions, few parents mentioned that their sons were not informed directly by their doctors, because they considered their own discussions adequate with their children. As for the information obtained by the parents, apart from the doctor who performed the operation and the internet, they sought information from every available source, including the pediatrician of the family, or a urologist.

#### 3.2.6. Sexual Life

Perception on the effect of the testicular prosthesis on sexual life

Whether or not they had already had the experience of sexual intercourse, certain recipients reported that they had or still have anxiety, stress, and doubts if the testicular prosthesis might affect their sexual life, if their partner might understand its presence, or if it would potentially produce discomfort to his partner or himself. Another group of recipients, however, mentioned that they knew that the testicular prosthesis, or the loss of one testis, would not affect their sexuality, perceiving that this is a non-important concern to deal with. R_09_ believed that the testicular prosthesis might compromise ejaculation.

All mothers considered that the prosthesis would positively affect the sexual life of their sons. They believed that it would help them to overcome the handicap of testicular absence compared to other normal males. A mother asked if her son should be careful with the prosthesis even during masturbation. Similarly, all fathers believed that though they had initially doubted, the testicular prosthesis would not negatively affect their sons’ sexual life.

Quality of sexual activity

Most of the recipients who had experienced intimate intercourse reported normal sexual activity. They mentioned that their erotic partners did not feel the presence of an artificial testis and were previously informed about its presence. An interesting observation is that if anxiety was produced, this occurred either during the first, or just before the initiation, of every sexual intercourse (R_01_). A participant reported that the feeling of his testicular prosthesis negatively affected masturbation, while R_13_ and R_15_ avoided touching the prosthesis during masturbation. R_09_ reported that he avoided sexual intercourse with his girlfriend because of fear of the tough and different feeling of the testis, and preferred paid sex, while R_13_ also paid for sex as he was investigating his new anatomical reality: “the first times I went to girls, you know, those who are paid for it, I told one of them to tell me if she noticed anything, like an experiment…she told me it was fine…I felt relief”. Only few parents discussed their sons’ sexual activity, reporting that it was anticipated for their age, and believing that in the future they would have a sexual life of a normal quality.

### 3.3. What About Those Who Denied Participation in the Study?

We considered it important to communicate our experience from the 13 phone calls who ended with refusal of participation in the study. They were six adults and seven minor recipients. The communication was performed with their parents, except once with the recipient himself. All conversations were emotionally intense. The outlined reasons included emotional and psychological concern as the recipient would revive the experience, discretion as that had been a painful intimate event, an overall effort to forget this experience, and embarrassment from the part of the recipient. The parental reactions were similar, expressing some negative feelings, mostly trying to protect their sons from reviving this experience. It is of note that a father reported that his son, even at the time of the contact, was unaware of the prosthesis, while another asked the researcher if his son would be able to have children and reported that he felt regret about the performance of the operation.

## 4. Discussion

In this study, we investigated the most important parameters which affected the QoL of males who had received a testicular prosthesis during their childhood. To our knowledge, this is the first qualitative study on such QoL, and one of the few with an integrated approach on recipients of a testicular prosthesis.

The starting point of this research presents an antithesis, an assumption, and a concern: any intervention on the male genitalia of a child produces negative feelings, such as discomfort, anxiety, and insecurity. On the other hand, the presence of a taboo situation such as a missing testis affects not only the patient himself but, like a contaminating disease, his parents, his companion, in other words his entire family and inner circle. Furthermore, young participants who have received a foreign body for the rest of their lives have been submitted to a metamorphosis of their most private part of their body, during the most tender and vulnerable age as boys or teenagers [24]. Finally, as this surgical intervention occurs in childhood or adolescence, the transition from the support of the pediatric specialties to those of adulthood may not always be smooth, resulting in unwanted psychological and intellectual consequences [25].

The small number of studies on patient satisfaction, sexual function, body image, and other aspects of QoL constitutes an obstacle to overcome [3,6,7,26]. According to a statement by Turek et al., there is a perception that the loss of a testis might reduce self-esteem; this has not been, however, sufficiently studied so far [3]. The application of QoL questionnaires showed quantifiable changes in several parameters of well-being in the prosthesis recipients. The researchers noticed statistically significant improvements in QoL in the pediatric population of the study compared to adults [3].

Zilberman et al., who performed a cross-sectional cohort study with questionnaires, also showed that the testicular prosthesis positively affected self-confidence [7]. Testicular prosthesis size, weight and position were the main parameters studied. The majority of the recipients considered the prosthesis comfortable and were soon used to its presence [7]. Regarding other QoL issues, the study population felt comfortable while undressing or spending time in female presence and none of them reported major problems during intercourse. Half of the recipients reported remarkable improvement in their body image and self-confidence. All would recommend the operation to a friend with a similar problem and would even undergo the operation again in case of complications [7].

Xylinas et al. performed a cross-sectional study on QoL of 72 testicular prosthesis recipients [26]. It is noted that 5% perceived their body image as worse than prior to surgery, and 80% considered their sexual activity unaffected. Most recipients considered the prosthesis worthwhile, while its sensation was the main cause of dissatisfaction (shape, size and cold effect) [26].

Araujo et al. performed a cross-sectional study regarding QoL and especially sexual function of 59 patients with orchiectomy, including 51 testicular prosthesis recipients [6]. An overall 96.1% satisfaction reflected a good perception. Esthetic comprehension of the implant and good physical and sexual life were the main parameters that were positively evaluated by the recipients [6].

The major topics that researchers mainly focused on in the literature were satisfaction for the appearance and feeling of the testicular implant. Concerns on size, position, weight, and shape have been thoroughly studied, with equivocal outcomes [27,28,29,30,31,32]. While there were studies which evaluated the testicular prosthesis as good or exceptional [9,31], other researchers reported significant levels of dissatisfaction, in association with position, feeling, or sexual intercourse [4].

In general, most studies evaluated the feeling of the testicular implant as positive [31,33,34,35,36]. Chronic pain, abnormal size, fixation compared to normal, cold effect, and higher position were reported as the main causes of discomfort [37]. Parents, in general, considered the testicular prosthesis appropriate and would recommend others with the same problem to proceed with such surgery [38].

Improved self-esteem, body and behavioral attractiveness, and positive feelings during sexual intercourse were considered as immediate and long-term outcomes of the testicular prosthesis [5,39]. Studies have reported that most recipients were satisfied when they were naked in front of both sexes, but when it came to intimate intercourse, most of them expressed anxiety [37,40]. By contrast, in other studies, the recipients reported an improved level of self-image and that their sexual life was not influenced [41]. While the psychological status of the recipients was significantly improved [42], there are studies which advocated the need to follow the patients for a long time period, to identify any long-term side effects [24,35].

Our study concurred with those which supported that the long-term effects were of utmost importance, and they would recommend their use to other potential recipients [5,6,28,32,34,43]. Nevertheless, there was variability in outcomes; patients of the same age, with the same preoperative disease, and with the same therapeutic results had completely different reactions. We may consider this heterogeneity as the result of the variability in characteristics such as age, elapsed time from surgery, social status, family issues and inherent characteristics.

Although we focused on each participant in detail, we might obtain a more comprehensive view by categorizing all our subjects into two groups according to their overall presence during the interviews. The first one included twelve of the participants who were fluent, willing, or expressing a positive attitude, especially those who had already experienced sexual intercourse or were in a relationship. Among them, the two patients with bilateral testicular prostheses were characterized by stoicism. The parents in this group were supportive, independently of their family, social, or financial status.

The second group included four participants who presented a more introverted behavior in the beginning and opened up gradually with time, however not at the level of the first group, i.e., insisting in brief responses of one or few words, or avoiding direct eye contact with the researcher. A common characteristic of these recipients was that there was a degree of tension in the family, usually among their parents. Three recipients of this group were single, while one had experienced sporadic relationships. There was one recipient in this group who certainly needed psychological assistance. His family influence was significantly negative, as the father continuously blamed himself for seeking delayed help after testicular torsion.

In both groups, the psychological condition and attitude of recipients and parents were better as the years passed from the time of surgery.

Another topic to mention was the direct provision of information by the doctor to the young recipient. It has been reported that incomplete supply of information is one of the main reasons that certain patients decline the implantation of a testicular prothesis [4,6,27,36]. The presence of the actual models and types of prostheses at the doctor’s office has also been suggested. It should be good for the candidate recipient to have the opportunity to see and touch various prosthesis items prior to operation, in order to achieve an immediate and thorough understanding of this novel, foreign object, and which has a new sense of touch and temperature, that will be inserted in his own most intimate body part [5,27]. The creation of brochures, webpages or smartphone applications with information about the testicular implant, adapted to younger patients, might be useful as well. Another point of interest is that we noticed the difference that while mothers focused mostly on the psychological well-being of their sons, the fathers cared more about their self-image and esthetic improvement.

Finally, some recipients expressed their satisfaction, as the interview conducted during this study triggered thoughts they had never had before. They said that the interview helped them to comprehend various aspects of the topic, previously unanswered.

This research has certain limitations. The study population consisted of a heterogeneous group of respondents in terms of age at the time of the interview. Another limitation might derive from the fact that we did not suggest, either preoperatively or prior to the interview, to the participants to seek assistance from a psychologist or a psychiatrist if required, thus missing the opportunity of assisting more effectively our patients and possibly obtaining useful information. Finally, the questionnaire used here was developed by the research team and was not validated. We acknowledge that incorporating an official assessment tool or questionnaire to evaluate the individual psychological profiles of the participants beyond our semi-structured questionnaire interview might have led to more comprehensive results.

## 5. Conclusions

Though testicular prosthesis implantation is considered a safe and of less technically challenging surgical operation, the timing of the surgery during the sensitive age of childhood and adolescence creates consequences which may affect the recipient for a lifetime. Thus, it should not be considered in a simplistic manner as a routine procedure. A thorough conversation should be conducted with the patient and his family, even with a mental health professional, from the very beginning. Available information should be provided according to their current age.

Finally, as the patients progress from the pediatric specialties to the adult medical services, they may experience a challenging transition. Many questions which might have arisen at a later age may remain unanswered, as reported in this study, in outcomes affecting his QoL. Therefore, a gentle transition with support and a smooth handover from the pediatric to adult medical workforce would be beneficial.

## Figures and Tables

**Table 1:** Questions addressed to the testicular prosthesis recipient and to his parents during the interview.

Questions Addressed to the Recipient
Are there any types of pain or discomfort impeding you from certain activities after the testicular prosthesis implantation?
Is there anything you believe that you are not able to do because of the testicular prosthesis?
Do you feel that you are affected by the comments of others, i.e., friends, classmates, in regard to your body image?
Did the testicular prosthesis modify your feelings and thoughts about yourself and your abilities?
How satisfied are you by yourself after the implantation of the testicular prosthesis?
Did you wish to discuss and share with your family, companion, friends, your feelings and thoughts, or certain practical issues on the testicular prosthesis implantation?
Could you describe the thoughts you had on your future sexual life before surgery?
Do you feel that the testicular prosthesis has affected your sexual life?
Do you consider that you have obtained from your doctor all the information you needed on the testicular prosthesis implantation?
Was the information you needed regarding the testicular prosthesis made available to you fully?
How does it feel to have the testicular prosthesis?
Did your doctor or your parents explain to you the reason for the testicular prosthesis?
What would you advise a young person to do, if he was in the same condition with you?
If you lost your testicular prosthesis, would you decide to have it implanted again?
**Questions addressed to the recipient’s parents**
Does he report any body complains or discomfort after the implantation of the testicular prosthesis?
Did he express before or after surgery any thoughts that his health might be compromised by the prosthesis?
Did he express his feelings, either positive or negative, regarding the testicular prosthesis?
Do you remember if he is affected by the comments of friends, classmates, or others in regard to his body image?
Is he able to communicate and share information, feelings and thoughts about himself after the testicular prosthesis implantation?
Has he expressed thoughts or concerns on his sexual life or intimate relationships?
Does he feel any restrictions in his life?
Did you discuss his problem with him, and did you ask him about the operation?
Do you believe that the doctor informed you in detail and answered all you questions on his health problem and the implantation of the testicular prosthesis?
Did you ever doubt his fertility and sexual life?
Are you afraid of something about his future?
Would you communicate and share with the family or others your own feelings and thoughts regarding his testicular prosthesis?
What would you advise a family in your own position today?

**Table 2:** Demographics and characteristics of the participants, who had received a testicular prosthesis, at the time of the study. Abbreviation: Testicular Prosthesis Recipient (R_00_).

Recipient Code	Age at Surgery (Years)	Age at Interview (Years)	Time from Surgery (Years)	Primary Testicular Disease	Occupation	Personal Life
R_01_	16	17	1	Testicular atrophy	Student	In relationship
R_02_	13	15	2	Testicular torsion	Student	Single
R_03_	13	17	4	Undescended testis	Student	Occasional relationships
R_04_	9	11	2	Undescended testis	Student	Single
R_05_	14	16	2	Testicular torsion	Student	Single
R_06_	14	23	9	Antenatal testicular torsion	University student	In relationship
R_07_	13	23	10	Testicular atrophy	University student	In relationship
R_08_	9	22	13	Testicular atrophy	Self-employed	Occasional relationships
R_09_	14	27	13	Undescended testis	University student	Occasional relationships
R_10_	14	22	8	Undescended testis	University student	Occasional relationships
R_11_	12	25	13	Undescended testis	University student	Occasional relationships
R_12_	8	14	6	Undescended testis	Student	Single
R_13_	14	21	7	Neonatal testicular torsion	University student	In relationship
R_14_	15	22	7	Anorchidism	University student	Single
R_15_	12	13	1	Testicular torsion	Student	Single
R_16_	14	18	4	Bilateral atrophic hypogonadism	University student	Single

**Table 3:** Demographics and characteristics of the recipients’ parents. Abbreviation: Mother (MR_00_), Father (FR_00_).

Parental Code	Age in Years	Family Status	Education Level	Occupation
M_R01_	47	Single	High school	Private employee
F_R01_	46	Divorced	High school	Private employee
M_R02_	44	Married	University	Self-employed
F_R02_	49	Married	University	Self-employed
F_R03_	51	Married	High school	Public employee
M_R04_	50	Married	High school	Private employee
F_R04_	49	Married	High school	Private employee
M_R05_	44	Married	High school	Housekeeper
F_R05_	54	Married	High school	Self employed
M_R12_	46	Widow	High school	Unemployed
M_R15_	48	Married	University	Public employee
F_R15_	50	Married	University	Self-employed

## Data Availability

Research data are available from the corresponding author upon reasonable request.

## Supplementary Materials

The following supporting information can be downloaded at https://www.mdpi.com/article/10.3390/children12060720/s1. Table S1: Domains investigated in the study and their subsections of special interest
