# Supplementary material for: Quality of Life of Adolescents and Young Adults After Testicular Prosthesis Surgery During Childhood: A Qualitative Study and Literature Review
Source: Children (Basel). 2025 May 31;12(6):720. doi: 10.3390/children12060720 (PMC12190729; doi:10.3390/children12060720)
Supplement: Supplementary file 1 [file children-12-00720-s001.zip › children-3661156-supplementary.pdf]

SUPPLEMENTARY MATERIAL

Table S1. Domains investigated in the study and their subsections of special interest.

**Domain**	**Codified subsections**
Physical health	Feeling of the testicular prosthesis
	Restriction of activities
	Perceptions of fertility
Mental health	Self-image
	Self-respect
	Feelings
Interpersonal relationships	Same-age peers
	Intimate partner
Family communication	Communicating with family members
	Communicating consent for surgery with the family
Access to information	Briefing with the doctor
	Search of information
Sexual life	Perception on the effect of the testicular prosthesis on sexual life
	Quality of sexual activity
